# Screening for Suitable Reference Genes for Quantitative Real-Time PCR in *Heterosigma akashiwo* (Raphidophyceae)

**DOI:** 10.1371/journal.pone.0132183

**Published:** 2015-07-02

**Authors:** Nanjing Ji, Ling Li, Lingxiao Lin, Senjie Lin

**Affiliations:** 1 State Key Laboratory of Marine Environmental Science, Xiamen University, Xiamen, Fujian 361005, China; 2 Department of Marine Sciences, University of Connecticut, Groton, Connecticut 06340, United States of America; Queen's University Belfast, UNITED KINGDOM

## Abstract

The raphidophyte *Heterosigma akashiwo* is a globally distributed harmful alga that has been associated with fish kills in coastal waters. To understand the mechanisms of *H*. *akashiwo* bloom formation, gene expression analysis is often required. To accurately characterize the expression levels of a gene of interest, proper reference genes are essential. In this study, we assessed ten of the previously reported algal candidate genes (*rpL17-2*, *rpL23*, *cox2*, *cal*, *tua*, *tub*, *ef1*, *18S*, *gapdh*, and *mdh*) for their suitability as reference genes in this species. We used qRT-PCR to quantify the expression levels of these genes in *H*. *akashiwo* grown under different temperatures, light intensities, nutrient concentrations, and time points over a diel cycle. The expression stability of these genes was evaluated using geNorm and NormFinder algorithms. Although none of these genes exhibited invariable expression levels, *cal*, *tub*, *rpL17-2* and *rpL23* expression levels were the most stable across the different conditions tested. For further validation, these selected genes were used to normalize the expression levels of ribulose-1, 5-bisphosphate carboxylase/oxygenase large unite (*HrbcL*) over a diel cycle. Results showed that the expression of *HrbcL* normalized against each of these reference genes was the highest at midday and lowest at midnight, similar to the diel patterns typically documented for this gene in algae. While the validated reference genes will be useful for future gene expression studies on *H*. *akashiwo*, we expect that the procedure used in this study may be helpful to future efforts to screen reference genes for other algae.

## Introduction

Harmful algal blooms (HABs) cause significant damages to marine ecosystems, local economies and human health [1–3]. *Heterosigma akashiwo* (Hada) Hada ex Y. Hara et Chihara is a HAB species within the class Raphidophyceae. This species is distributed worldwide, and is an eurythermal and euryhaline organism [4, 5]. *H*. *akashiwo* is notorious because its blooms have caused massive mortality of cultured finfish, but the mechanisms for ichthyotoxicity are not well resolved and remain controversial [6–9]. To understand how *H*. *akashiwo* forms blooms [10–13] and how the bloom kill fish, information on the molecular machinery or biochemical processes regulating growth and metabolism in this species is of great importance. Determining gene expression patterns under different environmental conditions is essential toward gaining such understanding.

Quantitative real-time reverse transcription polymerase chain reaction (qRT-PCR) is one of the most frequently-used technologies to study the expression patterns of genes, because it offers high sensitivity, specificity, reproducibility and accuracy [14, 15]. However, several variables associated with RNA samples may influence the accuracy of gene expression analysis, such as variations in RNA quantity and quality, enzymatic efficiency of reverse transcription and PCR amplification [16]. One common way to correct the biases caused by these factors is to normalize gene expression data against some properly established reference genes (housekeeping genes) [17–19]. Generally, an ideal reference gene should be expressed at stable levels in different tissues, under different treatments, or under different environmental conditions. Many genes have been used as reference genes, such as 18S ribosomal RNA (*18S*), glyceraldehyde-3-phosphate dehydrogenase (*gapdh*) and elongation factor 1α (*ef1*), simply because they have been shown to be appropriate in some model organisms. But, they have subsequently been found not to be always expressed at stable levels under different conditions in different species [20, 21]. Therefore, it is necessary to establish the suitability of the reference gene(s) for different species or specific types of conditions before use in gene expression studies. Until now, many studies have been carried out using or selecting reference genes in animals and plants [22–24] as well as algae (S1 Table). Yet no comprehensive screening for reference genes for *H*. *akashiwo* has been reported.

In this study, the stabilities in expression of ten candidate genes (Table 1), α-tubulin (*tua*), β-tubulin (*tub*), cytochrome c oxidase subunit II (*cox2*), 60S ribosomal protein L17-2 (*rpL17-2*), 60S ribosomal protein L23 (*rpL23*), calmodulin (*cal*), malate dehydrogenase (*mdh*), *18S*, *gapdh* and *ef1* in *H*. *akashiwo* were analyzed under four different experimental conditions. As a way to further validate the suitability of the most promising candidate genes identified, the two top-ranked reference genes for diel cycle studies were used to normalize rubisco large subunit (*HrbcL*) expression levels throughout a diel cycle. A set of four genes was found to be the most suitable as reference genes for all the four conditions we tested, while each condition had its own specific set of top-ranked reference genes.

**Table 1:** Candidate reference genes and *HrbcL* examined and their primers used in this study.

Gene symbol	Primer sequences (5'→3') Forward/Reverse	Amplicon length (bp)
*rpL17-2*	TACAGCATCAAGGACGAACCC	93
	GTTGTGGGCCACCTCTCTCA	
*rpL23*	GGTTTTCCCTGCTGTTGTCA	118
	GCCCTTCATTTCACCCTTGT	
*cox2*	CGATGTTTGGCTCTTTACGAC	130
	CGAATGAAGGAACTGCGATA	
*cal*	GCACCATTGACTTCCCTGAGT	113
	ACCGTTGCCATCCTTGTC	
*tua*	GCACCTTCTGCCTGGATAAC	174
	TGGTCTGGAACTCGGTCATA	
*tub*	CTTCAGACCCGACAACTTCG	123
	TCAGCCTCCTTTCTCACGAC	
*ef1*	AGTATGCCTGGGTGCTTGAC	105
	TGACGGTGTAGTGGAACTTGG	
*18S*	TGGTGGAGTGATTTGTCTGG	133
	CCCAACTTCCTTCGGTTAGTC	
*gapdh*	TACTGCGATGAGCCTTGTGTG	96
	GAACTTGGGGTTGAGGGAGA	
*mdh*	CCTCGTGATGTGAAGGTTGA	115
	GCGTGAGTTCCTCGATGTCT	
*HrbcL*	ACCACAACCTGGAGTAGACCC	182
	CGTATGCGATGTATGCGAAG	

## Materials and Methods

### *Heterosigma akashiwo* culture and treatment

*Heterosigma akashiwo* were maintained in a glass bottle with *f*/2 medium (without added silicate) prepared with 0.22 μm-filtered and autoclaved seawater (salinity 30 PSU). Stock cultures were kept at 20°C under a 14: 10 h light: dark cycle with an average photon flux of 100 ± 10 μE.m^-2^.s^-2^. For all experiments, cell concentrations were determined using a Sedgwick-Rafter chamber under the microscope.

For experiments, each treatment group was set up in triplicate. Control group was cultured in normal *f*/2 medium under the temperature and light conditions as described above. Temperature treatment included exposure to 10°C, 20°C and 30°C, respectively for 24 h. Light treatment consisted of exposure to 200 (high light), 100 (normal light) and 50 μE.m^-2^.s^-2^ (low light), respectively for 96 h. For nitrogen (N) and phosphorus (P) stress treatment, the cultures were treated as previously described [25] with minor modification. Briefly, the pre-treatment stock culture in *f*/2 medium was inoculated to low nutrient media that were either stoichiometrically low in N (181.5 μM NaNO_3_ and 36.3 μM NaH_2_PO_4_.H_2_O, with 5: 1 of N: P) or low in P (883 μM NaNO_3_ and 7.06 μM NaH_2_PO_4_.H_2_O, with 125: 1 of N: P) conditions. When these cultures were growing in the exponential phase, they were re-inoculated into fresh batches of their corresponding media. Cell concentration was monitored daily as described above, and when cultures were exhibiting exponential growth again, samples were collected. In addition, to examine the diel patterns of gene expression, the control group cultures were sampled, in which sampling started at 6 h (T6) after the onset of light period (T0), and 12 (T12), 18 (T18), 24 (T24), and 30 (T30) h after T0, throughout a 24-h diel cycle. Samples were collected by centrifugation at 3000 × rpm for 5 min at 4°C. The cell pellets were suspended in 1 mL TRIzol reagent (Invitrogen), and stored at -80°C until RNA extraction within a month.

### Total RNA extraction and cDNA synthesis

Total RNA was extracted as recently reported [26] and potential genomic DNA contamination was eliminated by incubating the RNA samples with RQ1 DNase (Promega). RNA was further purified by RNeasy Mini kit (Qiagen). RNA purity and concentration were measured by NanoDrop spectrophotometer (Theromo, Germany), and RNA integrity was evaluated by agarose gel electrophoresis.

The first-strand cDNA was synthesized from 300 ng of total RNA in a total volume of 20 μL using the ImProm-II reverse transcriptase (Promega) with random hexamer primer. The RNA template was first incubated with 0.5 μg of the primer at 70°C for 5 minutes, and then at 4°C for 5 minutes. Next, 4 μL ImProm-II 5 × reaction buffer, 4 μL MgCl_2_ (25 mM), 1 μL dNTP mix (10 mM each dNTP) and 1 μL ImProm-II reverse transcriptase were added and the mixture was incubated at 25°C for 5 minutes and then 42°C for 1 h. The reaction was terminated by incubating it at 70°C for 15 minutes. The cDNAs were diluted 1: 50 with nuclease-free water before use in subsequent experiments.

### Selection of candidate reference genes for study

A group of genes that have been used as reference genes in various algae were summarized in S1 Table. Based on the availability of the sequences from *H*. *akashiwo*, we selected eight (*tua*, *tub*, *18S*, *gapdh*, *rpL23*, *cal*, *ef1*, and *mdh*) from this list and added two others (*cox2* and *rpL17-2*) that are functionally related to *cox1* and *rpL19* in the list to achieve a set of ten candidate genes. The nucleotide sequences of *tua* (AY729829), *tub* (AY729817), *18S* (JX026930), *cox2* (GQ222228), and *gapdh* (AF319449), were available from GenBank database, and the others were identified from transcriptome data of *H*. *akashiwo* [27].

### Primer design and evaluation

The primers for qRT-PCR were designed using the Primer Premier 5.0 software and had estimated melt temperature of 57–60°C and amplicon lengths of 80–180 bp (Table 1). To check the specificity of the primers, regular PCR were run and all PCR products were examined on agarose gel for size, and then purified, cloned, and sequenced. For each primer, the PCR amplification efficiency (E) was calculated following E = [10^(-1/slope)^] × 100%, in which the slope were obtained from the standard curve generated from a serial dilutions of pooled cDNAs [28]. From the same dilution series correlation coefficient (R^2^) was also calculated.

### qRT-PCR

Using the cDNAs as the templates, qRT-PCR was conducted using the CFX96 Real-Time System (Bio-Rad, USA). Each PCR reaction was carried out in a total volume of 12 μL containing 6 μL of 2 × iQ SYBR Green supermix (Bio-Rad, USA), 375 nM of each primer, and 5 μL of 1: 50 diluted cDNA. The PCR program was composed of a denaturation step of 3 min at 95°C, followed by 40 cycles of 95°C for 10 s and 60°C for 32 s. In each run, negative controls were set up with ddH_2_O and RNA as templates, respectively. Each reaction had three technical replicates. At the end, to confirm primer specificity, all the PCR products were subjected to melting curve analysis.

### Gene stability analysis using geNorm and NormFinder

The geNorm [29] and NormFinder [30] software packages were employed to assess the stability of the expression levels of the candidate reference genes under the different experimental conditions. geNorm produces a stability measure (M) and by stepwise exclusion of the genes with the lowest stability creates a ranking of the tested genes (the lower the M value, the more stable the expression of the gene). The number of genes required for normalization of target gene expression also was estimated, and the normalization factor was calculated. NormFinder is a another program used to evaluate the candidate reference genes in given experimental design; it takes into consideration the intra- and inter-group variations and combines these results to estimate a reference gene stability value for each gene, avoiding influence caused by co-regulated candidate genes.

### Relative quantification of *HrbcL* gene expression

Ribulose 1, 5-bisphosphate carboxylase/oxygenase (*rbc*) is the key enzyme catalyzing CO_2_ fixation and is thus essential for photosynthetic organism [31]. Diel regulation of *rbc* transcription has been well documented in many photosynthetic organisms from cyanobacteria, algae, to higher plants [32]. Therefore, we attempted to characterize the diel expression patterns of *H*. *akashiwo rbc* large subunit (*HrbcL*) using the best reference genes (*rpL23* and *rpL17-2*) identified under diel cycle in the present study, as a way to further validate the selected reference genes. The sequence of *HrbcL* (EU168191) was obtained from GenBank, and primers were designed (Table 1). Relative expression levels of *HrbcL* were calculated by dividing the raw expression value of *HrbcL* for each sample by the normalization factor generated by geNorm.

### Statistical analyses

All statistical analyses were performed with SPSS (version 16.0). Variations in the Ct values between and within treatments as well as in the relative expression of *HrbcL* in *H*. *akashiwo* were analyzed using one-way ANOVA, and the level of significance was defined at *P* < 0.05.

## Results

### RNA quality, primer specificity and amplification efficiencies

RNA samples prepared from *H*. *akashiwo* all displayed good quality, with A260/A280 ratios ranging from 1.9 to 2.2, and A260/A230 ranging from 1.9 to 2.1. RNA integrity was confirmed on agarose gel electrophoresis, which showed two discrete bands, one (more abundant) 28S rRNA and another 18S rRNA. Specificities of primers were confirmed by the presence of a single band with the expected size on agarose gel electrophoresis (S1 Fig), the presence of a single peak in melting curve analysis after qRT-PCR (S2 Fig), and sequencing results (S1 File). No product was detected in negative control (ddH_2_O or RNA as template), indicating that there was no gDNA contamination in the RNA extracts, and the qRT-PCR results were thus reliable. The PCR efficiency (E) of the ten candidate reference genes and *Hrbc* ranged from 90.1%-101%, and the correlation coefficient (R^2^) ranged from 0.991 to 0.999, which were within the commonly reported range of qRT-PCR.

### Expression profiling of candidate reference genes

**Fig 1:**
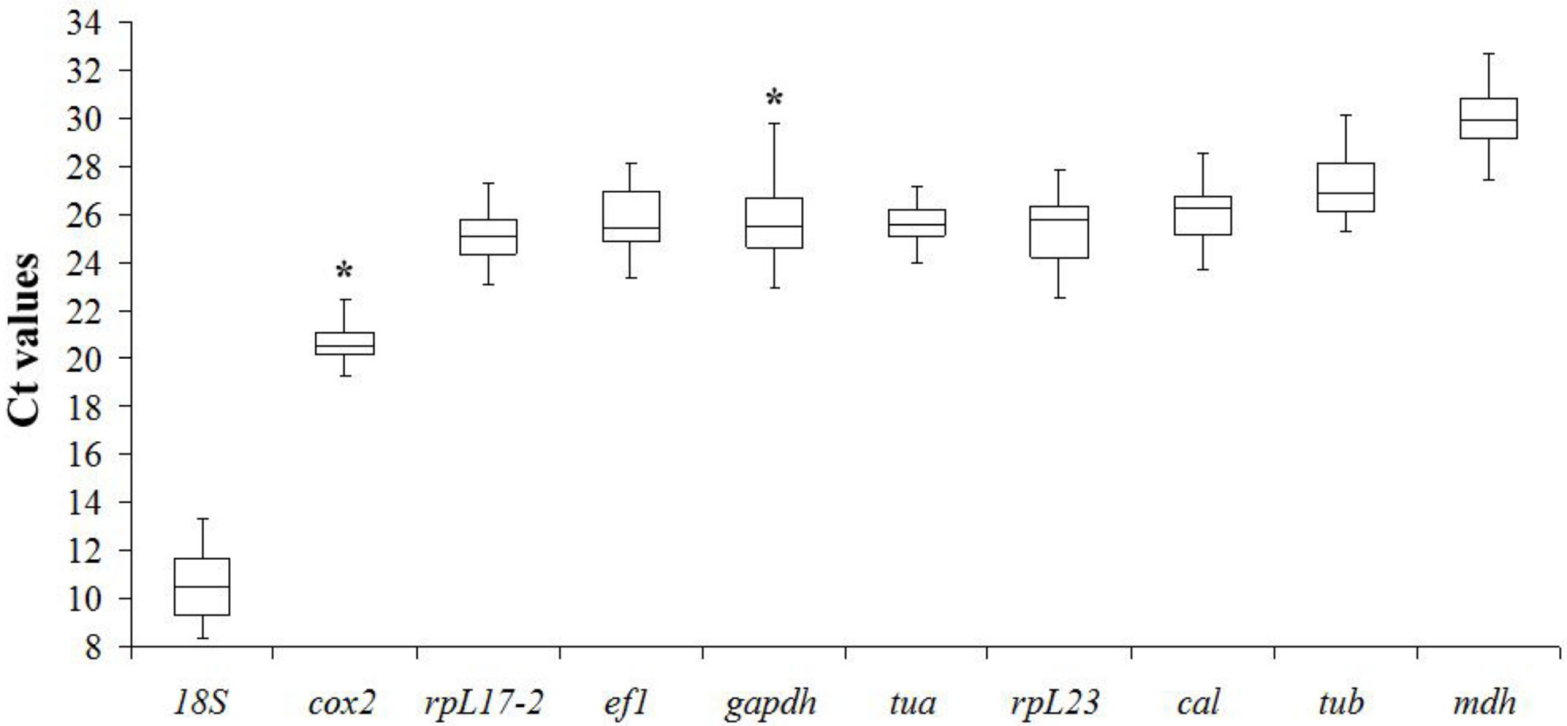
Ct values of candidate reference genes across conditions tested. Boxes show the median values (central lines), Q1 (lower outline) and Q3 (upper outline), and whiskers. The whiskers are set at 1.5 times IQR (interquartile range) above Q3 and 1.5 times IQR below the Q1. If the Maximun or Minimum values are outside this range, they are shown as outliers (*).

In order to investigate the relative expression levels of the ten candidate reference genes in *H*. *akashiwo*, the Ct values of these genes were calculated (S2 Table). The Ct median value of reference genes varied from 10.51 to 29.91, and most of the values were between 25.08 and 26.87 across all the samples (Fig 1). The *18S* gene showed the highest expression level with Ct value ranging from 8.33 to 13.32 in different samples, while *mdh* exhibiting the lowest expression level with Ct value ranging from 27.47 to 32.72. Based on the comparative ranges of Ct values, the smallest gene expression variation seemed to occur in *tua*, while *gapdh* seemed to be the most variable. However, a simple comparison of the raw Ct value is not sufficient to determine expression stability of the candidate reference genes; therefore, further analyses using geNorm and NormFinder software were conducted to provide more accurate results.

### Expression stability of candidate reference genes

**Fig 2:**
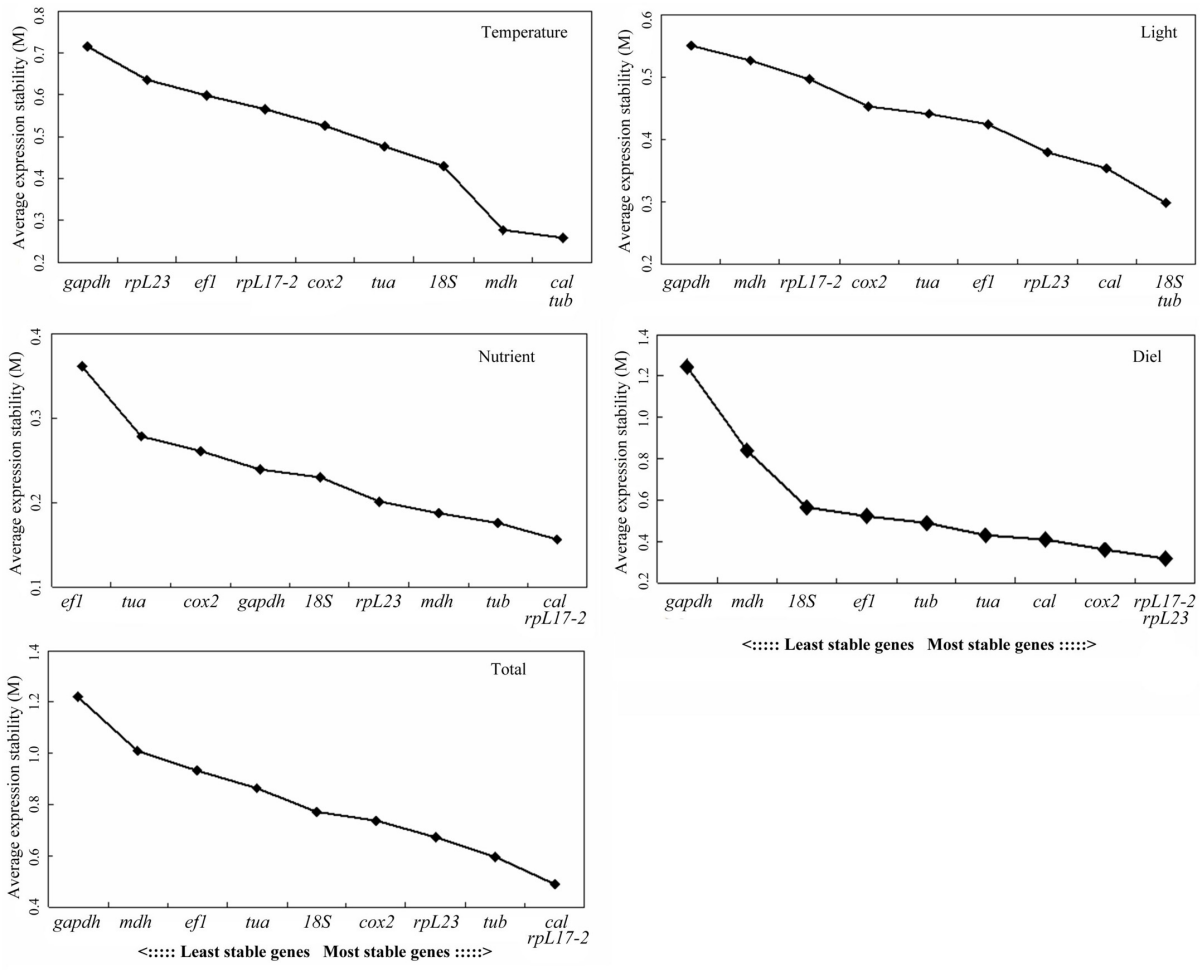
Gene expression stability and ranking of the candidate reference genes as calculated by geNorm. Average expression stability value (M) was calculated by stepwise exclusion of the least stable gene compared across different temperatures (Temperature), different light intensities (Light), different nutrient (N: P) ratios (Nutrient), different time points in the diel cycle (Diel), and the combination of all the samples (Total). In each plot, the least stable gene is on the left, and the most stable gene on the right.

Average expression stability values (M) were obtained using geNorm, and all candidate genes were ranked based on the M values (Fig 2). All ten genes investigated in this study showed M values below the threshold value 1.5, indicating that the expression levels of these genes were relatively stable under all the conditions we examined. By comparison, as shown in Fig 2, the most stable genes were *cal* and *tub* under temperature treatment, *18S* and *tub* under light treatment, *rpL17-2* and *rpL23* in the diel cycle samples, *cal* and *rpL17-2* under nutrient treatment and all treatments combined (M = 0.488) (Total). We found that *gapdh* was the least stably expressed under temperature treatment, light treatment, time points over the diel cycle, and total (M = 1.219), and *ef1* was the least stably expressed gene under nutrient treatment. In addition, our ANONA analysis showed that the variations in the *gapdh* and *ef1* Ct values were attributable to experimental treatments rather than inconsistencies between replicates, because the between group variance was much higher than the within group variance (S3 Table).

**Fig 3:**
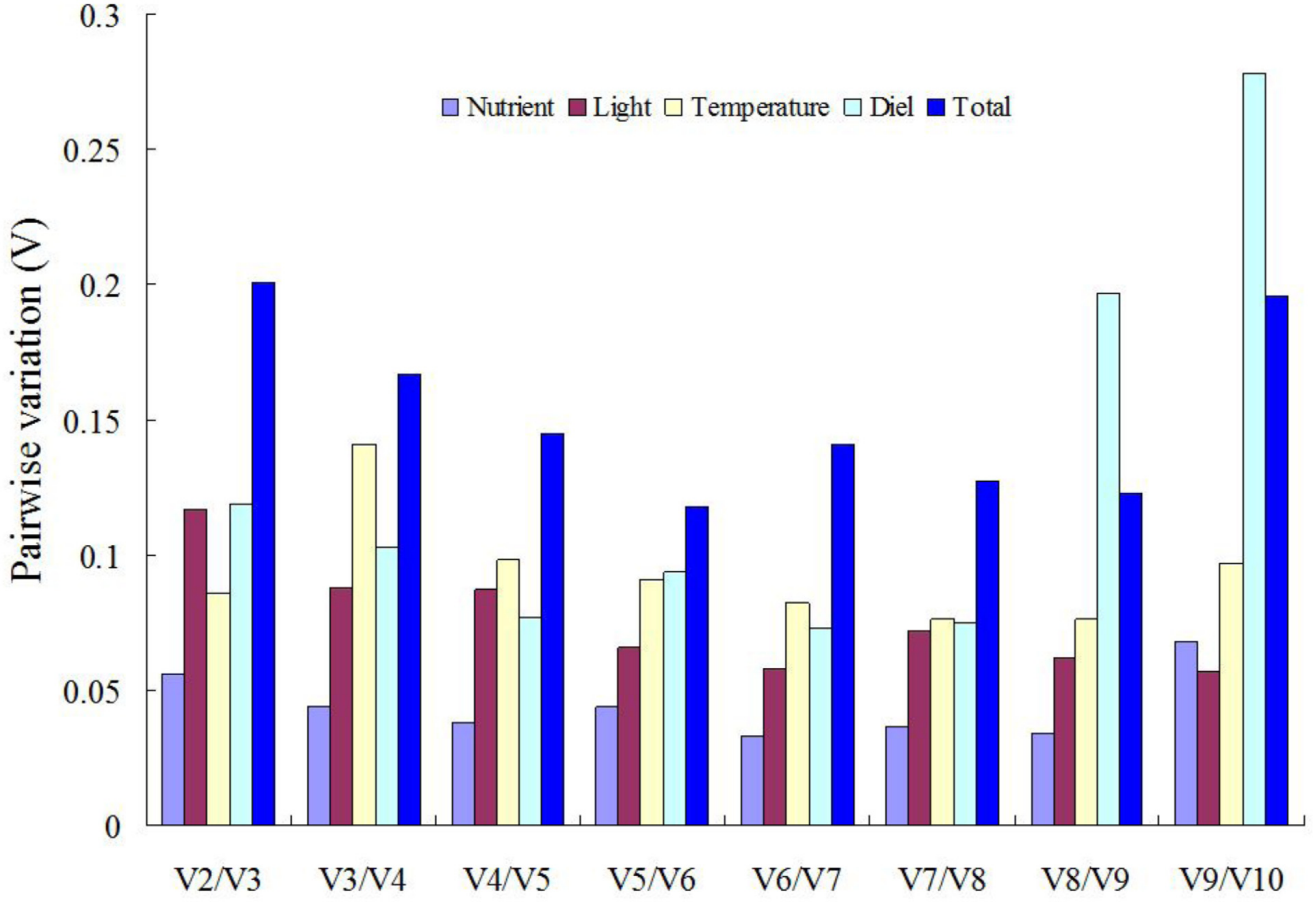
Determination of the optimal number of reference genes for normalizing gene expression (qRT-PCR) data. Pair-wise variation (V_n_/V_n+1_, where n represents number of genes) was analyzed between the normalization factors (NF_n_ and NF_n+1_) by geNorm software against all different conditions examined (nutrient, temperature, light, timing in the diel cycle and all these factors combined).

The geNorm software was also used to determine the optimal number of references genes required for accurate normalization, according to the pairwise variation (V_n_/V_n+1_) value. Vandesompele et al. (2002) proposed 0.15 as a V_n_/V_n+1_ threshold value, below which the inclusion of an additional control gene is not required [29]. For individual experimental treatment, V_2_/V_3_ value was 0.056, 0.117, 0.086 and 0.119 in nutrient, light, temperature and diel time point treatments, respectively (Fig 3). These results indicated that the inclusion of a third gene would not have significant effect for any of the four treatments, so the two most stable reference genes were sufficient for accurate normalization of gene expression under these conditions. When all the samples were considered together, V_2_/V_3_, V_3_/V_4_ and V_4_/V_5_ value were 0.201, 0.167 and 0.145, respectively. Combined with the result of stability ranking, the results showed that the four most stable reference genes, *cal*, *rpL17-2*, *tub* and *rpL23*, would be sufficient for accurate gene expression normalization for *H*. *akashiwo* under any combination of the four conditions.

For an independent assessment, the expression stabilities of reference genes were also ranked by NormFinder (Table 2). The most stable genes were *cal* for light and nutrient treatments, *rpL17-2* for temperature treatment, and *rpL17-2* and *rpL23* for diel cycle. When all the conditions were considered together, the same five most stable genes (*cal*, *rpL17-2*, *tub*, *rpL23*, and *cox2*) were identified by NormFinder as by geNorm, although *cox2* was ranked first by NormFinder and fifth by geNorm. NormFinder identified *gapdh* and *ef1* as the most unstable gene across all the conditions, which was consistent with the result from the geNorm.

**Table 2:** Expression stability values of the candidate reference genes calculated by NormFinder.

Temperature		Light		Nutrient		Diel		Total	
Ranking^a^	SV^b^	Ranking	SV	Ranking	SV	Ranking	SV	Ranking	SV
*rpL17-2*	0.247	*cal*	0.130	*cal*	0.096	*rpL23*	0.207	*cox2*	0.353
*cox2*	0.266	*rpL23*	0.176	*rpL23*	0.103	*rpL17-2*	0.230	*tub*	0.360
*mdh*	0.292	*18S*	0.226	*tub*	0.111	*cox2*	0.274	*cal*	0.361
*cal*	0.300	*tub*	0.234	*mdh*	0.142	*cal*	0.316	*rpL17-2*	0.370
*ef1*	0.319	*ef1*	0.285	*gapdh*	0.145	*tua*	0.405	*rpL23*	0.473
*18S*	0.374	*tua*	0.289	*rpL17-2*	0.149	*ef1*	0.570	*18S*	0.481
*tub*	0.375	*mdh*	0.302	*18S*	0.167	*tub*	0.576	*mdh*	0.489
*rpL23*	0.386	*cox2*	0.335	*cox2*	0.209	*18S*	0.588	*tua*	0.580
*tua*	0.390	*rpL17-2*	0.340	*tua*	0.234	*mdh*	0.848	*ef1*	0.689
*gapdh*	0.553	*gapdh*	0.367	*ef1*	0.408	*gapdh*	1.741	*gapdh*	1.020

^a^Ranking indicates the genes stability from the most stable to the least stable.

^b^SV represents stability value.

### Diel expression pattern of *HrbcL* and validation of the reference genes

**Fig 4:**
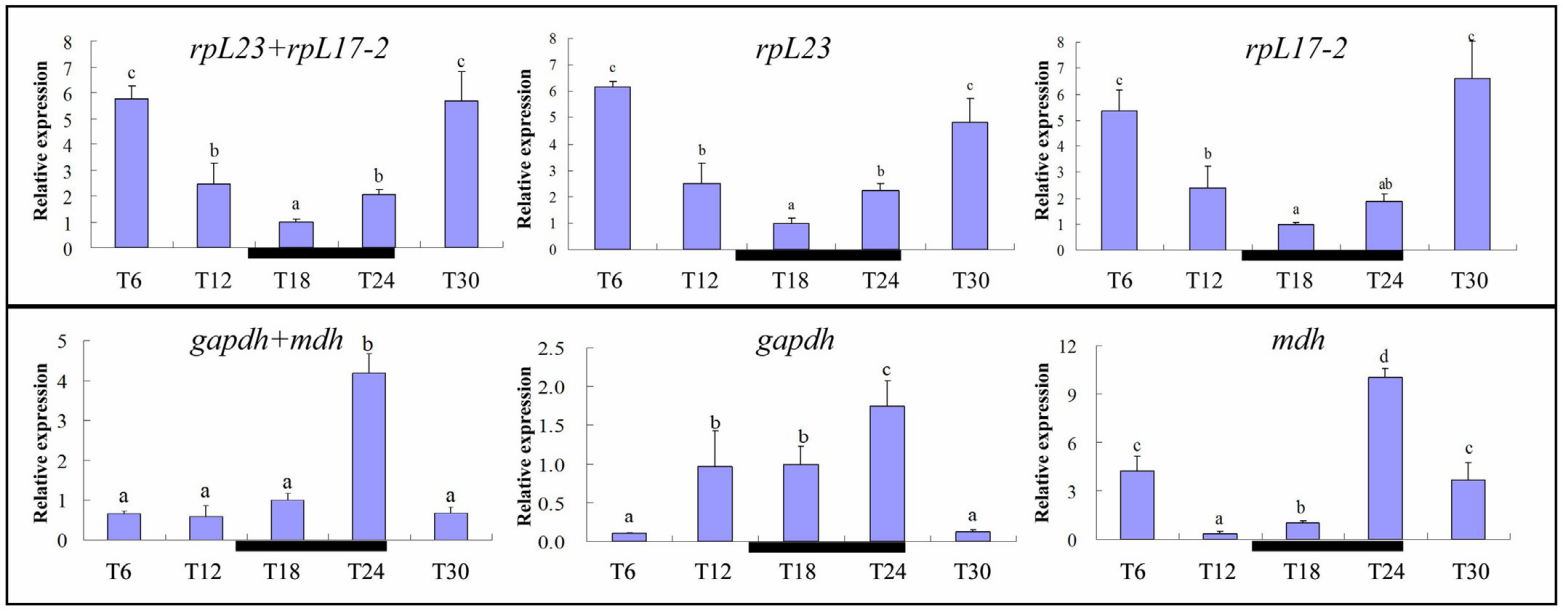
Diel pattern of *HrbcL* expression normalized against different reference genes. Relative expression values shown are mean fold changes compared to T18 samples, with error bars representing standard deviation (data were derived from triplicated biological (culture) samples, each with triple technical (qPCR) replicates). Different alphabets above the bars indicate significant differences at different time points at *P* < 0.05, while same letters within each plot indicate lack of significant difference. The black horizontal black bars indicate the dark period.

To evaluate the usefulness of the selected reference genes, we compared the expression pattern of *rbcL* in *H*. *akashiwo* (*HrbcL*) under diel cycle (sampled every six hours) using the most stable (*rpL17-2* and *rpL23*) and the least stable genes (*gapdh* and *mdh*) in the diel cycle among our ten candidate reference genes. When the most stable genes (*rpL17-2* and *rpL23*) were used in combination for normalization, the expression levels of *HrbcL* decreased rapidly between six hours after the onset of light (T6) and two hours before the end of the light period (T12), reaching the lowest value at four hours after the onset of the dark period (T18), and increased thereafter (Fig 4). A similar expression pattern was observed when either *rpL17-1* or *rpL23* was used alone for normalization. When the least stable genes (*gapdh* and *mdh*) were employed together, the normalized expression levels of *HrbcL* were not significantly different between time points T12 and T18, and entirely different expression patterns were obtained when either one of these two least stable genes was used for normalization (Fig 4).

## Discussion

Research on *H*. *akashiwo* has uncovered its life cycle and ecological characteristics, such as diel vertical migration [33], benthic stage [11], and strong ability to tolerate sudden salinity decrease [12], which may be linked to its ecological success. However, molecular mechanisms underlying these features [34] are poorly understood and need to be investigated. The qRT-PCR technology has been extensively used in the study of marine phytoplankton to understand transcriptional responses to physical stressors [35, 36], nutrient starvation [37], and diel cycle [26, 38, 39], and to detect the activity and modulation of many metabolic processes [39, 40]. As the accuracy of gene expression data is highly dependent on the selection of suitable reference genes for normalizing gene expression against experimental variations, most of those studies used reference genes for the normalization. However, only a small faction of those reference genes has been systematically evaluated to ensure they are suitable for the species under investigation.

In this paper, we conducted careful evaluation on expression stability of ten candidate reference genes, eight of which have been used in previous studies on various organisms. We chose to investigate the effects of temperature, light, and nutrient conditions because they are the most common environmental variable influencing marine phytoplankton. Diel cycle was also examined because sampling for molecular analysis, particularly on research cruises, often occurs at different times of the day. Two widely used analysis programs, geNorm and NormFinder, were used to ensure reliability of gene expression stability assessment. Due to their distinct algorithms, slight differences were observed when the rankings of the candidate reference genes from these two programs were compared. For example, under temperature variations, *rpL17-2* was ranked to be the most stable by NormFinder, whereas *cal* and *tub* were ranked as the most suitable candidate reference genes by geNorm (Fig 2, Table 2). Corresponding different results have also been reported and discussed in many previous studies [22, 23, 41]. However, both analysis programs produced the same top five most stable genes: *cal*, *rpL17-2*, *tub*, *rpL23*, and *cox*2. Therefore, these five can be considered the “short list” of reference genes for common transcriptional studies in *H*. *akashiwo*. Based on the below-threshold pairwise variation, V_4_/V_5_ value (0.145) and the high frequencies at which these genes were ranked favorable across the four separated sets of conditions, *cal*, *rpL17-2*, *tub* and *rpL23* are particularly preferable for use under different environmental stress conditions or diel cycle of *H*. *akashiwo*. Picking common top-ranked genes from multiple analysis programs should give high confidence about the selection of the reference genes.

Even for specific environmental conditions or treatments, the picking common-gene approach can be also helpful for identifying best reference genes available. For temperature treatment, for instance, geNorm identified *cal* and *tub* as the most stable genes, followed by *mdh*, *18S*, and others (Fig 2), whereas NormFinder ranked *rpL17-2* as the best reference gene, followed by *cox2*, *mdh*, *cal*, and others (Table 2). Based on the ranking orders, *cal* and *mdh* would be the best choices among the ten examined presently for temperature effect studies. This result agrees with the previous studies on *cal* and *mdh* genes in algae. The calmodulin gene has been identified as a stable reference gene in the dinoflagellate *Symbiodinium* sp. under temperature stress [35], while *mdh* was shown to be relatively stable in the dinoflagellate *Prorocentrum minimum* across many experimental conditions (heat shock, toxic chemical exposures and different life stages) [42].

By the same way, we found *cal* and *tub* were sufficiently stable to be used for normalizing gene expression under light and nutrient treatments. The beta-tubulin gene has also been selected as reference gene in studies of gene expression in the chlorophyte macroalga *Ulva linza* [43] and the diatom *Pseudo-nitzschia multistriata* [44].

For diel cycle samples, both geNorm and NormFinder identified *rpL23* and *rpL17-2* as the ideal reference genes, and the pairwise variation V_2_/V_3_ of 0.119 indicated that these two ribosomal protein genes could be used in combination for normalization (Fig 3). Recently, various ribosomal proteins genes have frequently been identified as suitable reference genes in algae and other organisms. *rpS*30 (ribosomal protein small subunit 30S) gene has been selected as the most stable gene among twelve candidate reference genes compared throughout a diel cycle [38]. *rpL19* (60S ribosomal protein L19) and *rpL23* were validated as reference genes in the chlorophytes *Chlamydomonas* sp. (for freezing condition) [45] and *Volvox carteri* (for different cell types) [46], respectively.

The GAPDH gene (*gapdh*) has been considered to be a suitable reference gene for quantifying gene expression in many algal species under different conditions, such as *Prorocentrum donghaiense* under diel cycle [26], *Alexandrium catenella* in P-limited conditions [47] and *Chlamydomonas* sp. under different light treatment [48]. However, in our study, *gapdh* was ranked as one of the least stable genes under different treatments and in all samples combined, indicating that it is not suitable as a reference gene for *H*. *akashiwo* under our experimental conditions. In agreement of our result, the mRNA and protein levels of *gapdh* have been reported to be regulated by light in other algae [38, 49]. The variability of *gapdh* expression level also has been increasingly recognized for other types of organisms [29, 50, 51].

Like *gapdh*, *18S* is commonly used as a reference gene [52], and it has been used to design probes for quantifying the abundance of *H*. *akshiwo* in field samples [53]. In the present study we found that *18S* was a moderately stable gene across all conditions we examined, although it appeared to be a suitable reference gene under light treatment (Fig 2). Compared with other genes (mRNA transcripts) the Ct median value of *18S* was much lower (Fig 1), indicating that the abundance of *18S* transcript was much higher (~1000 folds that of *cox2* and ~700,000 folds that of *mdh*). The high abundance of *18S* compared to mRNA transcripts (target genes) makes it difficult to reliably subtract the background baseline value in qRT-PCR data analysis [29, 54]. In addition, *18S* rRNA content may be affected by nutrient stress and vary over the diel cycle [25, 55]. Therefore, we recommend that *18S* not be selected as a reference gene for qRT-PCR in *H*. *akashiwo*.

Applying the candidate reference genes to normalizing a well-characterized target gene would provide further validation for the reference genes. We chose to assess the expression profile of *rbcL* in *H*. *akahsiwo* (*HrbcL*) under a diel cycle because *rbcL* mRNA abundance is known to exhibit strong diel rhythm in many algal species [32]. We observed a similar diel rhythm in *HrbcL* expression whether the most stable genes reference genes (*rpL23* and *rpL17-2*) for diel cycle were used individually or in combination (Fig 4) to the diel pattern reported for many algae [32, 56], i.e. expression level being the highest around the middle of the light period and lowest in the middle of the dark period. Many previous reports also have shown that the expression patterns of target genes showed similar trends when either single or combined most stable reference genes were used [28, 57]. This further verifies that *rpL23* and *rpL17-2* are suitable reference genes for *H*. *akashiwo* gene expression studies under diel (light dark) cycle. When the expression levels of *HrbcL* were normalized with the least stable genes (*gapdh*, *mdh*) for diel cycle, either singly or in combination, different diel patterns of *HrbcL* expression were observed, either by comparison to each other, to previously reported patterns, or to patterns when *rpL23* and *rpL17-2* were used for normalization (Fig 4). Clearly, *gapdh* and *mdh* are not suitable reference genes for gene expression studies in *H*. *akashiwo* under diel cycle. These results demonstrate that the use of reference genes without validation risks misinterpretation of results.

## Conclusion

To our knowledge, this work is the first study to evaluate candidate reference genes for gene expression analysis in *H*. *akashiwo* under different environmental conditions (temperature, nutrient and light) and different time points over the diel cycle. After careful assessment using qRT-PCR combined with statistical analysis based on geNorm and NormFinder, our results show that *cal* and *tub* are good reference genes for gene expression studies under different light and nutrient conditions, *rpL17-2* and *rpL23* for diel cycle studies, *cal* and *mdh* for varying temperature conditions. Our results also lead us to conclude that if used in combination *cal*, *tub*, *rpL17-2* and *rpL23* are suitable reference genes for gene expression analysis under all the four different experimental conditions we examined, and because these conditions represent the most common environmental or sampling factors, they may be applicable to most field studies. The identification of the suitable reference genes in this study will facilitate future studies on gene expression in *H*. *akashiwo* to improve our understanding on the molecular mechanisms of bloom formation.

## Supporting Information

S1 Table Summary of reference genes that had been tested or used in algal research. (DOC)

S2 Table Ct values of candidate reference genes examined in this study. (XLS)

S3 Table Effects of different treatments on the Ct value of two least stable genes (*gapdh* and *ef1*). (DOC)

S1 Fig Agarose gel electrophoresis results of ten candidate reference genes and *HrbcL* PCR products. (TIF)

S2 Fig Melting curves of ten candidate reference genes and *HrbcL*. (TIF)

S1 File Sequence results of ten candidate reference genes and *HrbcL*. (FASTA)
