# Supplementary material for: Screening for Suitable Reference Genes for Quantitative Real-Time PCR in Heterosigma akashiwo (Raphidophyceae)
Source: PLoS One. 2015 Jul 2;10(7):e0132183. doi: 10.1371/journal.pone.0132183 (PMC4489630; doi:10.1371/journal.pone.0132183)
Supplement: S1 Table — (DOC) [file pone.0132183.s001.doc]

Table S1. Summary of reference genes that had been tested or used in algal gene expression studies.

| Organism | Reference genes^a^ | Treatments | Reference |
| --- | --- | --- | --- |
| **Pyrrophyta** |  |  |  |
| *Symbiodinium* sp. | *sam*, *rpS4* | Thermal | Rosic et al., 2010 ^b^ |
|  | c*yc*, *sam*, *rpS4*, *cal,* *cox1* | Thermal and Light |  |
|  | *β-act*, *pcna* | Light | Boldt et al., 2008 |
| *Prorocentrum donghaiense* | *gapdh,* *cal* | Diel cycle | Shi et al., 2013 |
| *Prorocentrum minimum* | *tua, mdh* | Copper or temperature | Guo et al., 2012 ^b^ |
|  | *tua, ubq* | Life stage |  |
| *Alexandrium catenella* | *gapdh* | P limitation | Zhang et al., 2014 |
| *Karenia brevis* | *cob,* *gapdh* | P limitation | Lin et al., 2012 |
| **Chlorophyta** |  |  |  |
| *Chlamydomonas* sp. ICE-L | *ef1* | Diel cycle; Light, salinity or UV | Mou et al., 2014 ^b^ |
|  | *gapdh* | Light |  |
| *Chlamydomonas reinhardtii* | *18S* | Light | Fischer et al., 2007 |
|  | *rpL19* | Freezing | Liu et al., 2012 |
| *Dunaliella salina* | *β-act* | Salt | Chen et al., 2011 |
| *Ulva fasciata* | *18S* | Chemical | Hsu et al., 2012 |
| *Ulva linza* | *tub* | Salinity and UV | Dong et al., 2012 ^b^ |
|  | *h2* | Temperature and desiccation |  |
|  | *18S* | Light |  |
| *Nannochloropsis* sp. | *β-act*, *tua* | Light, diel cycle or temperature | Cao et al., 2012 ^b^ |
| *Volvox carteri* | *18S,* *ef1* | Developmental stages or stress | Kianianmomeni et al., 2013 ^b^ |
|  | *rpL23*, *tbp* | Different cell types |  |
| **Bacillariophyta** |  |  |  |
| *Skeletonema costatum* | *18S* | Chemical | Chung et al., 2008 |
| *Eolimna minima* | *β-act* | Cd exposure | Tiam et al., 2012 |
| *Ditylum brightwellii* | *tua,* *β-act* | Thermal or Metal | Guo et al., 2013 ^b^ |
| *Thalassiosira pseudonana* | *β-act,* *ubl* | P or Fe limitation | Alexander et al., 2012 ^a^ |
| *Phaeodactylum tricornutum* | *rpS30*, *tbp* | Diel cycle | Siaut et al., 2007 ^b^ |
| *Pseudo-nitzschia multistriata*  *P. arenysensis* | *tua*, *tub*  *cdkA* | Different growth phases;  Nitrogen starvation; | Adelfi et al., 2014 ^b^ |
| **Raphidophyta** |  |  |  |
| *Heterosigma akashiwo* | *gapdh,* *18S* | Nitrate starvation | Coyne et al., 2010 |
| **Rhodophyta** |  |  |  |
| *Pyropia yezoensis* | *β-act*, *ef1*, *eIf4A,*  *ubq*, *ef1*, *eIf4A* | Abitotic stress  Developmental stages | Kong et al., 2014 ^b^ |
| *Gracilaria lemaneiformis* | *gapdh*, *ef1*, *β-act* | Temperature | Ding et al., 2014 |
|  | *gapdh,* *β-act* | Different life generations |  |
| **Phaeophyta** |  |  |  |
| *Laminaria japonica* | *β-act* | Temperature and Salinity | Fu et al., 2009 |
| *Ectocarpus siliculosus* | *tua*, *ubl,* *ef1* | Diel cycle or Chemical | Le Bail et al., 2008 ^b^ |

^a^Gene names, *18S*=small subunit (40S) ribosomal RNA; *β-act*=beta-actin; *cdkA=*cyclin dependent kinase A; *cal*=calmodulin; *cob*=cytochrome B; *cox* *1*=cytochrome C oxidase 1; *cyc*=cyclophilin; *ef1*=elongation factor 1; *eIf4A*=translation initiation factor 4A; *gapdh*= glyceraldehyde-3-phosphate dehydrogenase; *h*2=histone 2; *rpL19*=large subunit (60S) ribosomal protein 19; *rpS4*=small subunit (40S) ribosomal protein 4; *sam*=s-adenosylmethionine synthetase; *tbp*=TATA-box binding protein; *tua*=alpha-tubulin; *tub*=beta-tubulin; *ubl*=ubiquitin ligase; *ubq*=ubiquitin.

^b^Study that mainly focused on selection of reference gene in the tested alga.

**References**

1. Rosic NN, Pernice M, Rodriguez-Lanetty M, Hoegh-Guldberg O. Validation of housekeeping genes for gene expression studies in *Symbiodinium* exposed to thermal and light stress. Mar Biotechnol. 2011; 13: 355-365.

2. Boldt L, Yellowlees D, Leggat W. Measuring *Symbiodinium* sp. gene expression patterns with quantitative real-time PCR. Proceedings of the 11th ICRS, 7-11 July 2009, Ft. Lauderdale, Florida, pp. 118-122.

3. Shi X, Zhang H, Lin S. Tandem repeats, high copy number and remarkable diel expression rhythm of form II RuBisCO in *Prorocentrum donghaiense* (dinophyceae). PLoS One. 2013; 8: e71232.

4. Guo R, Ki J-S. Evaluation and validation of internal control genes for studying gene expression in the dinoflagellate *Prorocentrum minimum* using real-time PCR. Eur J Protistol. 2012; 48: 199-206.

5. Zhang C, Lin S, Huang L, Lu W, Li M, Liu S. Suppression subtraction hybridization analysis revealed regulation of some cell cycle and toxin genes in *Alexandrium catenella* by phosphate limitation. Harmful Algae. 2014; 39: 26-39.

6. Lin X, Zhang H, Huang B, Lin S. Alkaline phosphatase gene sequence characteristics and transcriptional regulation by phosphate limitation in *Karenia brevis* (Dinophyceae). Harmful Algae. 2012; 17: 14-24.

7. Mou S, Zhang X, Miao J, Zheng Z, Xu D, Ye N. Reference genes for gene expression normalization in *Chlamydomonas* sp. ICE-L by quantitative real-time RT-PCR. J Plant Biochem Biot. 2014: 1-7.

8. Fischer BB, Krieger-Liszkay A, Hideg E, Snyrychova I, Wiesendanger M, Eggen RI. Role of singlet oxygen in chloroplast to nucleus retrograde signaling in *Chlamydomonas reinhardtii*. FEBS letters. 2007; 581: 5555-5560.

9. Liu C, Wu G, Huang X, Liu S, Cong B. Validation of housekeeping genes for gene expression studies in an ice alga *Chlamydomonas* during freezing acclimation. Extremophiles. 2012; 16: 419-425.

10. Chen H, Lao YM, Jiang JG. Effects of salinities on the gene expression of a (NAD+)-dependent glycerol-3-phosphate dehydrogenase in *Dunaliella salina*. Sci Total Environ. 2011; 409: 1291-1297.

11. Hsu YT, Lee TM. Modulation of gene expression of carotene biosynthesis-related protein by photosynthetic electron transport for the acclimation of intertidal macroalga *Ulva fasciata* to hypersalinity and excess light. Physiol Plantarum. 2012; 144: 225-237.

12. Dong M, Zhang X, Chi X, Mou S, Xu J, Xu D, et al. The validity of a reference gene is highly dependent on the experimental conditions in green alga *Ulva linza*. Curr Genet. 2012; 58: 13-20.

13. Cao S, Zhang X, Ye N, Fan X, Mou S, Xu D, et al. Evaluation of putative internal reference genes for gene expression normalization in *Nannochloropsis* sp. by quantitative real-time RT-PCR. Biochem Bioph Res Co. 2012; 424: 118-123.

14. Kianianmomeni A, Hallmann A. Validation of reference genes for quantitative gene expression studies in *Volvox carteri* using real-time RT-PCR. Mol Biol Rep. 2013; 40: 6691-6699.

15. Chung CC, Hwang S-PL, Chang J. Nitric oxide as a signaling factor to upregulate the death-specific protein in a marine diatom, *Skeletonema costatum*, during blockage of electron flow in photosynthesis. Appl Environ Microb. 2008; 74: 6521-6527.

16. Tiam SK, Feurtet-Mazel A, Delmas F, Mazzella N, Morin S, Daffe G, et al. Development of q-PCR approaches to assess water quality: effects of cadmium on gene expression of the diatom *Eolimna minima*. Water Res. 2012; 46: 934-942.

17. Guo R, Lee M-A, Ki J-S. Normalization genes for mRNA expression in the marine diatom *Ditylum brightwellii* following exposure to thermal and toxic chemical stresses. J Appl Phycol. 2013; 25: 1101-1109.

18. Alexander H, Jenkins BD, Rynearson TA, Saito MA, Mercier ML, Dyhrman ST. Identifying reference genes with stable expression from high throughput sequence data. Front Microbiol. 2012; 3: 385.

19. Siaut M, Heijde M, Mangogna M, Montsant A, Coesel S, Allen A, et al. Molecular toolbox for studying diatom biology in *Phaeodactylum tricornutum*. Gene. 2007; 406: 23-35.

20. Adelfi MG, Borra M, Sanges R, Montresor M, Fontana A, Ferrante MI. Selection and validation of reference genes for qPCR analysis in the pennate diatoms *Pseudo-nitzschia multistriata* and *P*. *arenysensis*. J Exp Mar Biol Ecol. 2014; 451: 74-81.

21. Coyne KJ. Nitrate refuctase (NR1) sequence and expression in the harmful alga *Heterosigma akashiwo* (Raphidophyceae). J Phy. 2010; 46: 135-142.

22. Kong F, Cao M, Sun P, Liu W, Mao Y. Selection of reference genes for gene expression normalization in *Pyropia yezoensis* using quantitative real-time PCR. J Appl Phycol. 2014: 1-8.

23. Ding Y, Sun H, Zhang R, Yang Q, Liu Y, Zang X, et al. Selection of reference gene from *Gracilaria lemaneiformis* under temperature stress. J Appl Phycol. 2014: 1-8.

24. Fu W, Yao J, Wang X, Liu F, Fu G, Duan D. Molecular cloning and expression analysis of a cytosolic Hsp70 gene from *Laminaria japonica* (Laminariaceae, Phaeophyta). Marine Biotechnol. 2009; 11: 738-747.

25. Le Bail A, Dittami SM, de Franco PO, Rousvoal S, Cock MJ, Tonon T, et al. Normalisation genes for expression analyses in the brown alga model *Ectocarpus siliculosus*. BMC Mol Biol. 2008; 9: 75-83.
