# Supplementary material for: Screening for Suitable Reference Genes for Quantitative Real-Time PCR in Heterosigma akashiwo (Raphidophyceae)
Source: PLoS One. 2015 Jul 2;10(7):e0132183. doi: 10.1371/journal.pone.0132183 (PMC4489630; doi:10.1371/journal.pone.0132183)
Supplement: S3 Table — (DOC) [file pone.0132183.s003.doc]

Table S3: Effects of different treatments on the Ct value of two least stable genes (*gapdh* and *ef1*).

| Treatments | Genes | Source | SS | *df* | MS | *F* | *p*-value |
| --- | --- | --- | --- | --- | --- | --- | --- |
| Temperature | *gapdh* | Between Group | 9.638 | 2 | 4.819 | 32.821 | 0.001 |
|  |  | Within Group | 0.881 | 6 | 0.147 |  |  |
| Light | *gapdh* | Between Group | 5.137 | 2 | 2.568 | 12.470 | 0.007 |
|  |  | Within Group | 1.236 | 6 | 0.206 |  |  |
| Nutrient | *ef1* | Between Group | 5.368 | 2 | 2.684 | 21.939 | 0.002 |
|  |  | Within Group | 0.734 | 6 | 0.122 |  |  |
| Diel | *gapdh* | Between Group | 64.339 | 4 | 16.085 | 70.297 | 0.000 |
|  |  | Within Group | 2.288 | 10 | 0.299 |  |  |

SS=Sum of Squares; MS=Mean Square.
