# Supplementary figures and images for: Screening for Suitable Reference Genes for Quantitative Real-Time PCR in Heterosigma akashiwo (Raphidophyceae)
Source: PLoS One. 2015 Jul 2;10(7):e0132183. doi: 10.1371/journal.pone.0132183 (PMC4489630; doi:10.1371/journal.pone.0132183)

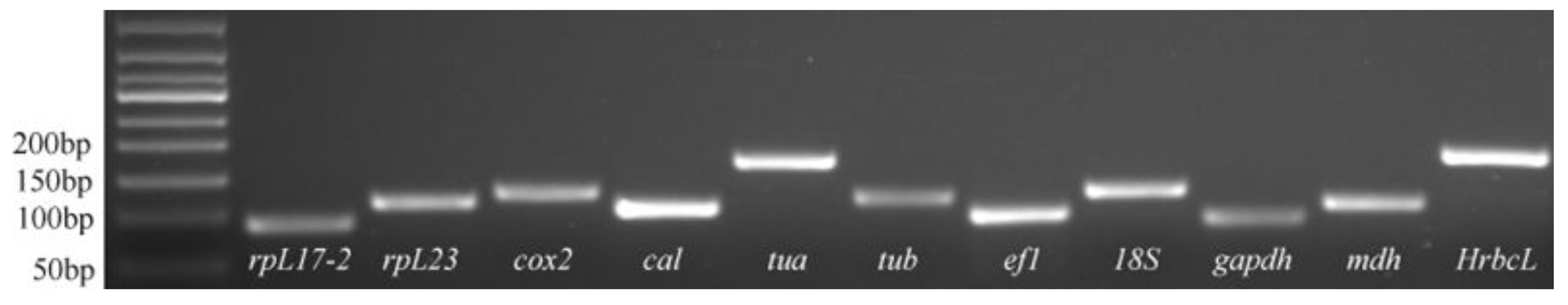

Supplement: S1 Fig — (TIF) [file pone.0132183.s004.tif]

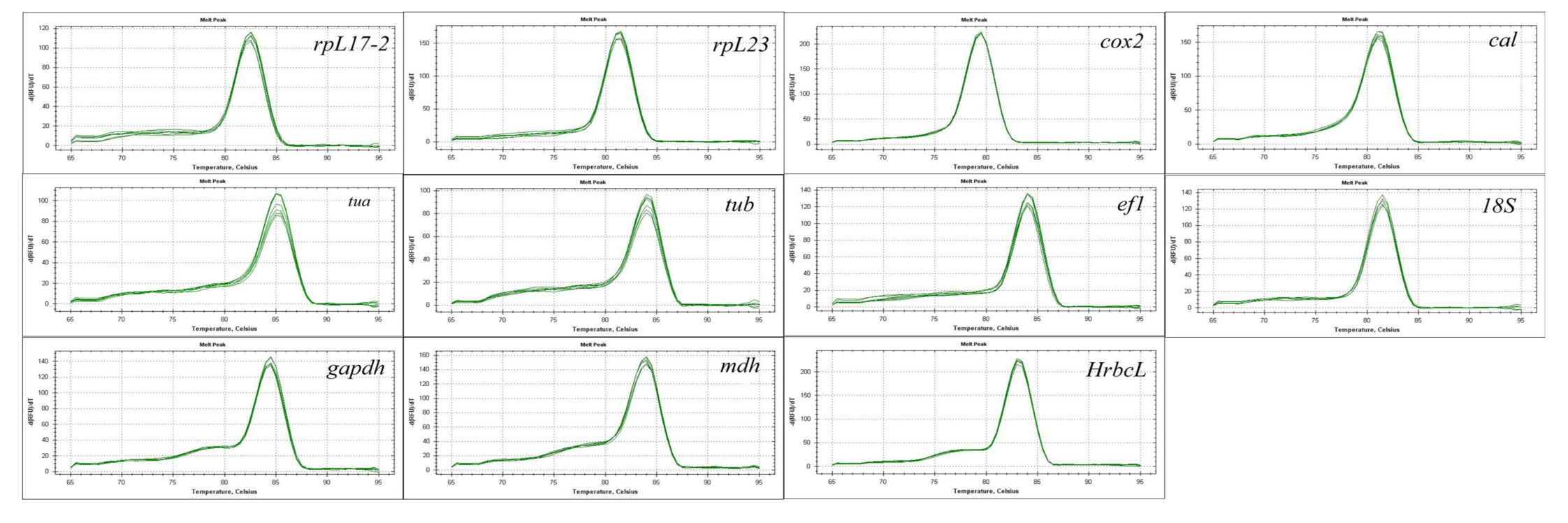

Supplement: S2 Fig — (TIF) [file pone.0132183.s005.tif]
